# Neoadjuvant versus Concurrent Androgen Deprivation Therapy in Localized Prostate Cancer Treated with Radiotherapy: A Systematic Review of the Literature

**DOI:** 10.3390/cancers15133363

**Published:** 2023-06-27

**Authors:** Rodrigo Cartes, Muneeb Uddin Karim, Steven Tisseverasinghe, Marwan Tolba, Boris Bahoric, Maurice Anidjar, Victor McPherson, Stephan Probst, Alexis Rompré-Brodeur, Tamim Niazi

**Affiliations:** 1 Department of Radiation Oncology, McGill University, Montreal, QC H3A 0G4, Canada; 2 Department of Radiation Oncology, McGill University, Gatineau, QC J8V 3R2, Canada; 3 Department of Radiation Oncology, Dalhousie University, and Nova Scotia Health Authority, Sydney, NS B1P 1P3, Canada; 4 Department of Urology, McGill University, Montreal, QC H3A 0G4, Canada; 5 Department of Nuclear Medicine, McGill University, Montreal, QC H3A 0G4, Canada

**Keywords:** prostate cancer, radiotherapy, ADT sequencing, neoadjuvant ADT, concurrent ADT

## Abstract

**Simple Summary:**

Multiple randomized trials have highlighted the importance of combining Androgen Deprivation Therapy (ADT) and Radiotherapy in the management of localized intermediate (IR) and high-risk (HR) prostate cancer (PCa). Recent trials have shown that the moment of initiation of ADT seems to be clinically relevant. The purpose of our review was to compile the available evidence on behalf of the combination of RT and ADT, focusing on the sequencing of both modalities to provide recommendations on the optimal timing to start the hormonal therapy.

**Abstract:**

Background: There is an ongoing debate on the optimal sequencing of androgen deprivation therapy (ADT) and radiotherapy (RT) in patients with localized prostate cancer (PCa). Recent data favors concurrent ADT and RT over the neoadjuvant approach. Methods: We conducted a systematic review in PubMed, EMBASE, and Cochrane Databases assessing the combination and optimal sequencing of ADT and RT for Intermediate-Risk (IR) and High-Risk (HR) PCa. Findings: Twenty randomized control trials, one abstract, one individual patient data meta-analysis, and two retrospective studies were selected. HR PCa patients had improved survival outcomes with RT and ADT, particularly when a long-course Neoadjuvant-Concurrent-Adjuvant ADT was used. This benefit was seen in IR PCa when adding short-course ADT, although less consistently. The best available evidence indicates that concurrent over neoadjuvant sequencing is associated with better metastases-free survival at 15 years. Although most patients had IR PCa, HR participants may have been undertreated with short-course ADT and the absence of pelvic RT. Conversely, retrospective data suggests a survival benefit when using the neoadjuvant approach in HR PCa patients. Interpretation: The available literature supports concurrent ADT and RT initiation for IR PCa. Neoadjuvant-concurrent-adjuvant sequencing should remain the standard approach for HR PCa and is an option for IR PCa.

## 1. Introduction

Androgen deprivation therapy (ADT) plays an important role in the management of advanced prostate cancer (PCa). Hodges and Huggins’ [1] seminal studies established the pre-eminent role of ADT in suppressing metastatic hormone-sensitive PCa cells. Subsequently, ADT was shown to be beneficial in localized PCa, synergistically with radiotherapy (RT) both in the definitive and post-operative setting. While several studies have sought to assess the benefit of treatment intensification with novel ADT agents [2], ADT sequencing with RT has until recently not been thoroughly explored.

In terms of rationale, neoadjuvant hormone therapy (NAHT) was thought to improve outcomes by better controlling occult micrometastases and circulating tumor cells. Neoadjuvant (NA) ADT allows better downstaging of disease. Furthermore, ADT-induced cytoreduction permits better tumor targeting and may improve clearance of high radiation isodoses from surrounding organs at risk [3,4]. However, concurrent and adjuvant hormone therapy (C-AHT) is thought to better sensitize cells to RT by inhibiting androgen-receptor-mediated DNA repair following ionizing radiation [5].

Preclinical data have pointed to the possible benefits of both approaches. Murine models have demonstrated that tumor growth was better impeded with castration before RT. Indeed, a lower radiation dose was needed to attain tumor control in mice orchiectomized before rather than after RT [6]. However, radiation-induced up-regulation of the androgen receptor (AR) and tumor-related neo-angiogenesis may be prime targets for the synergistic effects of C-AHT [7,8].

Although historically NAHT may have been preferred over the concurrent initiation of ADT with RT, contemporary data seem to favor the latter approach [9,10]. Therefore, we conducted a systematic review of the literature to assess if Adjuvant over the Neoadjuvant initiation of ADT offers clinical advantages in terms of metastases-free survival and/or overall survival for IR and HR PCa treated with RT and ADT.

## 2. Materials and Methods

We conducted a systematic review of the literature pertaining to the combination and sequencing of ADT and RT. Different approaches were reviewed including NAHT, Concurrent Hormone therapy (CHT), and Adjuvant Hormone Therapy (AHT) in the context of definitive RT for localized intermediate risk (IR) and high-risk (HR) PCa. This systematic review was conducted in accordance with the PRISMA (Preferred Reporting Items for Systematic Reviews and Meta-Analyses) guidelines and registered in PROSPERO (ID: CRD42023415370).

PubMed (1946–July 2022), Embase (1974–12 July 2022), and Cochrane databases (2005–2022) were queried. In each of these databases, we used a combination of the terms “Radiotherapy, Prostate Cancer, Neoadjuvant, Concurrent Hormone Therapy, Androgen Deprivation Therapy, Radiotherapy, Sequencing, Hormonal therapy”. Table 1 summarizes the keywords and search results per database.

**Figure 1:**
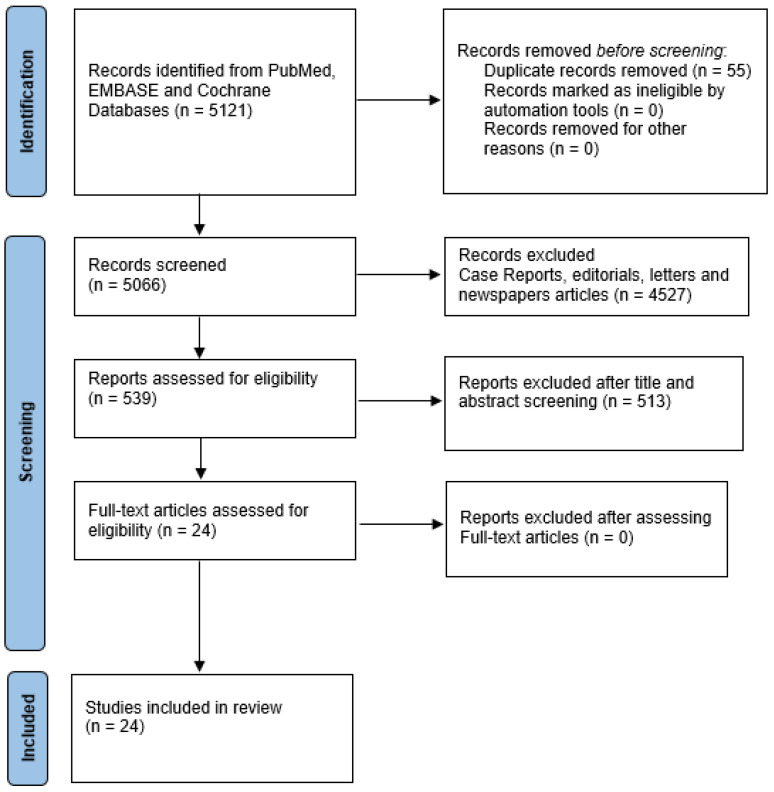
PRISMA Flow Diagram of included records.

Initially, we identified 5121 articles in PubMed, EMBASE, and Cochrane. We limited the query to papers that were written in English, or which had an available English translation. One study on Cochrane, 166 studies on PubMed, and 372 articles on EMBASE were identified. Case reports, editorials, letters, and newspaper articles were excluded from the database research. All the identified abstracts were reviewed by each of the authors and an independent selection of articles was made. The selection criteria were to include articles assessing the combination of definitive RT and HT in IR and HR PCa and studies addressing the sequence of HT and RT in the same clinical setting. After removing duplicates, 24 articles were then selected during a consensus meeting. One meta-analysis, twenty randomized controlled trials (RCT), one abstract of an RCT, and two retrospective studies were fully examined by the authors. Figure 1 shows the PRISMA flow diagram of the selected and included records. Regular consensus meetings were held to discuss and extract information, as well as to resolve all disagreements between authors related to study selection and inclusion.

## 3. Results

Our systematic review comprehensively assessed all major clinical trials evaluating the role and optimal sequencing of ADT combined with RT. Table 2 summarizes the relevant selected RCTs. We identified 19 RCTs comparing different combinations and durations of RT combined with hormone therapy in IR and HR PCa patients. Table 3 compiles studies that specifically assessed ADT sequencing with curative RT, in either the neoadjuvant, concurrent, and adjuvant setting. We identified five relevant studies, including retrospective analyses, phase III trials, and a meta-analysis. The main objective of this review was to synthesize the currently available evidence to elucidate an optimal sequencing of ADT with RT in IR and HR PCa.

Weller et al. published a retrospective analysis of a prospectively maintained prostate cancer registry from the Cleveland Clinic. The study identified 515 patients treated with dose-escalated EBRT (78 Gy at 2 Gy/fx or 70 Gy at 2.5 Gy/fx) and 6 months of ADT. The aim was to compare and contrast ADT sequences. Group 1 received NA-CHT (n = 311) starting 2–3 months before RT and then CHT for a total of 6 months. Group 2 received adjuvant-only HT, beginning the last day of RT for 6 months in total (n = 204). Radiation was delivered to the prostate alone. The majority of patients had HR (46.6%) or unfavorable IR disease (21.2%). These patients were grouped together and analyzed in the high-risk group. The median age was 69 years, stage T3 was present in 12.4%, prostate-specific antigen (PSA) > 20 ng/mL occurred in 25.6%, and Gleason 8+ was present in 22.1%. At 10 years of follow-up, no difference was found between ADT sequences for bRFS (63% vs. 61%, *p* = 0.98), DMFS (80% in both, *p* = 0.60), and OS (65% vs. 67%, *p* = 0.98). When analyzing HR and IR patients separately, no difference in biochemical control was identified between sequences. The multivariate analysis revealed that T stage, PSA, Gleason, and risk group were independent predictive factors for biochemical failure and distant metastases. The authors concluded that the synergy between RT and ADT was independent of sequencing and that it was not necessary to delay definitive treatment [49].

Lee et al. published a retrospective study of 11,491 patients with high-risk prostate cancer treated with definitive RT and ADT from the National Cancer Database (NCDB). The authors assessed the impact of NAHT administered 42–90 days before RT vs. C-AHT starting 14 days before RT to 84 days after RT initiation. Total ADT duration however was not reported. The study found that the median OS was significantly improved in the NAHT group (111.4 vs. 108.9 months). This also remained statistically significant in the multivariate analysis [50]. While compelling, given the retrospective nature of the trial, this finding remains hypothesis-generating.

The NRG/RTOG 9413 trial was a two-by-two factorial study comparing NA-Concurrent HT or Adjuvant-only HT with either WPRT or PORT. The study’s primary endpoint was progression-free survival. Patients in the NA-CHT arm started ADT two months before RT and stopped at the end of radiation. Patients in the AHT arm received ADT for 4 months after completion of radiation. In total, this trial enrolled and randomized 1322 patients. At 10 years, NA-CHT improved PFS compared to AHT in the patients receiving WPRT (28.4% (95% CI 23.3–33.6) vs. 19·4% (14.9–24.0)). Conversely, NA-CHT did not improve PFS compared to AHT in patients receiving PORT (23.5% (18.7–28.3) vs. 30·2% (25.0–35.4)). The authors concluded that in patients with IR and HR PCa, NA-CHT plus pelvic RT improved progression when compared to pelvic RT with AHT or prostate-only RT with NA-CHT. However, long-term follow-up revealed that there was increased grade ≥ 3 gastrointestinal toxicity in approximately 7% of patients receiving WPRT. However, after analysis, the authors found an interaction between hormonal therapy and RT. They, therefore, concluded that WPRT should be avoided without NAHT [51].

The Ottawa 0101 was a Phase III trial that assessed NA-CHT versus C-AHT. Inclusion criteria encompassed patients with newly diagnosed localized IR and HR prostate cancer, Gleason score ≤ 7, clinical stage T1b to T3a, and PSA < 30 ng/mL. Patients on NA-CHT received 6 months of ADT starting 4 months before RT, followed by two months of CHT (arm A). Patients on C-AHT received 6 months of ADT starting simultaneously with RT (Arm B). In total, 432 patients were recruited and randomly assigned to either the NA-CHT or C-AHT arm. Respectively, 96.3% (n = 207) and 94.5% (n = 205) had IR PCa in NAHT and CHT groups. WPRT was not used in this trial. No significant difference was found between groups for bRFS (80.5% vs. 87.4%, *p* = 0.10) or OS rates (76.4% vs. 73.7%, *p* = 0.70) at 10 years. CSM rates at 10 years were 2% and 1.9% in arms A and B, respectively. Local progression-free survival rates (LPFS) for arms A and B were 92.2% (95% CI, 86.5% to 98.3%) and 90.4% (95% CI, 83.1% to 98.2%). Metastasis free-survival (MFS) was 94% (95% CI, 90.0% to 98.3%) for arm A and 95.1% (95% CI, 91.5% to 98.9%) for arm B. There was no significant difference between the treatment groups on the log-rank test (*p* = 0.60 for both LPFS and MFS). Similarly, there were no significant differences between groups for either GI (2.5% vs. 3.9%) or GU toxicity (2.9% vs. 2.9%), nor did any RT-related grade ≥ 3 occur. The authors thus concluded that both neoadjuvant and concurrent short-term ADT with RT were reasonable standards of care, given there was no difference between outcomes [9].

Recently, Spratt et al. published an individual patient data meta-analysis on the sequencing of ADT and RT for localized prostate cancer. Overall, 1065 patients from RTOG 9413 and Ottawa 0101 trials were included and stratified in the NA-CHT (n = 534) or AHT/C-AHT (n = 531) group. The exact number of patients with IR and HR PCa was not reported. However, baseline characteristics suggested the cohort comprised primarily IR patients. Higher risk features were also identified. Gleason 8–10 was reported in 16.9%, cT3-T4 was present in 19.6% and a PSA greater than 20 was identified in 35.6%. With a median follow-up of 14.9 years, the adjuvant approach was found to significantly improve PFS (29% vs. 36%, HR 1.25 [95% CI, 1.07 to 1.47], *p* = 0.01), BF (sHR 1.37), DM (sHR 1.40) and MFS (sHR 1.17 [95% CI, 1.00 to 1.37], *p* = 0.050). Late grade 3 GU and GI toxicity did not differ between groups. Hence, these results favored AHT/C-AHT over the NA-Concurrent ADT approach for localized PCa [10].

Bias Assessment

The risk of bias in the included studies was assessed. The main limitation of Weller’s study is the risk of selection bias given its retrospective design, despite data coming from a prospectively maintained registry. Although the population was heterogeneous, the authors considered this in the statistical analysis. Another strength of this study is the homogeneity in the treatment volumes and length of ADT, which may have reduced the risk of bias.

In the same way, Lee’s retrospective analysis of the NCDB is subjected to selection bias. The NCDB does not report on the ADT length, therefore, it cannot be excluded that patients in the NAHT group received systematically longer periods of hormone therapy. Furthermore, differences in performance status, access to treatment, or other patient-related factors not systematically reported in the NCDB could not be considered in the analysis. Additionally, the population in this study and the treatment interventions were heterogeneous. The authors accounted for immortal time bias by excluding patients that lived less than 6 months.

On the other hand, the RTOG 9413 randomized design reduced the risk of selection bias. However, the patient population was heterogeneous as modern risk stratification was not used to enroll patients. Moreover, the interaction seen between treatment volume and timing of ADT was not considered in the design of this trial, and as stated by the authors, some of the reported associations could be just by chance, or in the context of a statistical bias associated with the factorial design of the trial. Additionally, the primary endpoint, Progression Free Survival, is mainly driven by PSA control, and therefore not necessarily clinically meaningful.

Similarly, the Ottawa 0101 also had a reduced risk of selection bias by using a randomized design. However, patients with Gleason 7 were not stratified according to the primary Gleason score, leading to possible unbalance between treatment arms and precluding assessment of a differential treatment effect. Nonetheless, the population was less heterogeneous than in other trials, including mainly IR PCa, and there was nearly complete adherence to protocol-specified therapy.

Finally, Spratt’s Meta-analysis was conducted on individual patient data from two randomized trials designed to assess the sequence of ADT and RT. This reduced the risk of selection bias, but not completely excluded it as the analysis was not conducted prospectively. The patients treated with pelvic RT were excluded to harmonize the populations of RTOG 9413 and Ottawa 0101. However, this could potentially have led to intervention bias if pelvic RT is considered standard in HR PCa. Additionally, there was heterogeneity in the interventions between the included studies, especially on the duration of ADT and the exact moment of initiation of the hormone therapy.

## 4. Discussion

The addition of ADT to RT was initially studied in HR patients as an adjuvant therapy. The RTOG 85-31 demonstrated significantly better LC, MFS, and OS when combining indefinite AHT with RT [11,12,13]. Subsequently, several other studies explored the addition of Concurrent ADT to low-dose RT. The addition of a 4 to 6-month course of NA-Concurrent ADT demonstrated better PCSM and OS at 10 years in the RTOG 86-10 and TROG 96-01 studies respectively, when compared to RT alone [14,15,16,24,25]. These benefits were not seen when hormone therapy was given for a shorter course of 3 months [24,25]. Longer ADT courses of 36 months, as in the EORTC 22863 trial, of Concurrent-AHT with RT, have also improved OS compared to RT alone [17,18,19]. On the other hand, the addition of RT to long-course ADT in the TAP 32, PR3/MRC PR07, and SPCG-7 trials has shown an improvement in PCSM and OS respectively [41,42,44,45,47,48].

When comparing short and long courses of ADT combined with low-dose RT in HR PCa patients, the optimal duration has always favored long-term ADT. Crook et al. reported that 8 months of NA-HT had better DFS in HR disease than a 3-month course. Similarly, the EORTC 22961 found inferior OS at 5 years with 6 vs. 36 months of Concurrent-AHT [22,23,26]. The same trend was seen with NA-Concurrent-AHT, the most common sequence used in the trials addressing the ideal duration of ADT when combined with RT. The RTOG 92-02 and DART-01/05 GICOR found that 28 months of ADT improved OS compared to 4 months in HR PCa and that these results were still clinically meaningful with long-term follow-up, even in the dose-escalated era [20,21,28,29]. The TROG 03-04 reported better PCSM when combining RT with 18 months of NA-Concurrent-AHT compared to 6 months of NA-Concurrent ADT, with no change in outcomes with the addition of Zoledronic Acid [30,31,32]. Finally, the Canadian PCS-IV study, comparing an 18- vs. 36-month course of NA-Concurrent-AHT, suggested that the optimal duration of ADT should be ≥18 months in HR PCa treated with RT [27]. Hence, these trials have firmly established that long-term ADT combined with RT is the standard of care for HR PCa. Although not designed to address the question of sequencing, these studies mainly used a short period of NAHT, followed by concurrent and long-term AHT.

Subsequent phase III trials in IR PCa evaluated short-term ADT in combination with RT. The general trend was towards improved disease control and PCSM. However, a consistent OS benefit was not seen across studies. D’Amico et al. and the RTOG 94-08 trial reported that 4 to 6 months of NA-concurrent hormones with low-dose RT improved PCSM [33,34,35,36,37]. In D’Amico’s study, OS was worse in the RT alone group than in the subgroup of men with minimal or no cardiovascular comorbidities [33,34,35]. In the RTOG trial, there was a survival benefit in the subgroup of men with IR PCa treated with RT + ADT, though this was no longer seen at 15 years of follow-up [36,37]. In the dose-escalated era, the EORTC 22991 failed to demonstrate a DM or OS benefit at 10 years with 6 months of Concurrent-AHT and RT, although better DFS was observed [38,39]. On the other hand, the Canadian PCS-III study found that 6 months of NA-Concurrent ADT with low-dose or dose-escalated RT improved PCSM compared to high-dose RT alone. There were no differences between 70 Gy and 76 Gy in the combination arms [40]. Finally, the published abstract of the RTOG 0815 comparing DE-RT alone with or without 6 months of TAS reported better PCSM in the TAS-arm, with no differences in OS [43]. Again, as in HR PCa, the preferred sequence in the IR trials included a short course of NAHT followed by CHT with RT and a short AHT period, but there was no direct comparison of ADT sequence-related outcomes.

Our systematic review identified five studies that aimed specifically at ADT sequencing: two Phase III trials (RTOG 94-13 [51] and Ottawa 0101 [9]) that were analyzed separately have seeded conflicting views, a meta-analysis of these two studies [10], and two large retrospective analyses from the Cleveland Clinic [49] and the NCDB [50].

The NRG/RTOG 94-13, through its factorial design, aimed to address the impact of treatment volume (pelvis vs. prostate only) and ADT timing (NAHT vs. AHT) in IR and HR PCa. Unexpectedly, a significant interaction between treatment volume and ADT timing was identified in this study [51]. Indeed, a PFS advantage favoring NAHT over AHT was observed in the patients receiving pelvic RT but not in those receiving prostate-only RT. This trial included an inherently heterogeneous population and the design did not contemplate a statistical interaction between the treatment field and hormonal sequencing [51]. Moreover, this study had a few notable shortcomings, given that HR PCa patients were undertreated with suboptimal radiation doses relative to current standards (70.2 Gy), short-term ADT and not having pelvic RT in all patients, although the latter may still be debatable. Thus, in retrospect, optimal risk stratification-based management may have provided clearer insights into the impact of ADT sequencing.

Alternately, the Ottawa 0101 trial mainly included intermediate-risk PCa patients treated with dose-escalated prostate-only RT and 6 months of ADT, 2 months of concurrent with either 4 months of NHT or 4 months of AHT. At 10 years, no survival difference was identified. A non-significant trend to improved biochemical control was seen with the concurrent/adjuvant approach (80.5% vs. 87.4%, *p* = 0.10) [9]. This study follows the current standard clinical practice for RT dose, volumes, and ADT duration in intermediate-risk patients. However, the distinction between favorable and unfavorable intermediate-risk subgroups and their apparent differential benefit from short-term ADT was not considered in the design of the Ottawa trial. Even when no prospective trials have directly assessed this issue, patients with unfavorable IR disease have been reported to have increased rates of BF, DM, and CSM [52]. The addition of a short course of ADT has been shown to improve these outcomes. Arguably, ADT only helps to improve biochemical control in favorable IR patients with no reported impact on metastases or mortality [52,53,54]. Hence, it is debatable if an unfavorable intermediate-risk-only patient population would have garnered a greater benefit with concurrent-adjuvant sequencing.

Spratt et al. combined the RTOG 94-13 and Ottawa trial in an individual patient data meta-analysis, excluding the patients who received pelvic RT. Although risk stratification was not reported, high-risk patients were estimated to represent at least a third of the patient population through its baseline characteristics. Contrary to the individual analysis of both trials, the meta-analysis found that NHT was significantly associated with poorer PFS (HR 1.25), higher biochemical failure (sHR 1.37), more distant metastases (sHR 1.40) and lower MFS (HR 1.17) at 15 years. This study currently provides the highest quality of evidence on ADT and RT sequencing [10]. As per its inclusion criteria, these findings can be mainly extrapolated to intermediate-risk disease. Though, subgroup heterogeneity marring the findings for high-risk groups cannot be completely excluded, given that this analysis was not reported. Again, several confounding factors including low RT dose in the RTOG trial, exclusion of the pelvis and suboptimal ADT duration in high-risk patients, and inclusion of favorable IR disease should be considered when interpreting this study. Additionally, IR PCa is commonly treated with 2 months of NAHT, CHT, and AHT, respectively [55]. Both, NA-CHT and C-AHT sequencing have shown benefits in IR PCa when combined with DE-RT for 6 months. The Canadian PCS-III study reported better PCSM with 6 months of NA-CHT when compared to DE-RT alone [40]. On the other hand, the EORTC 22991 showed improved DFS with 6 months C-AHT versus DE-RT alone [38,39]. Consequently, to obtain the benefits of both approaches, AHT is given despite a NA course of hormone therapy has been already received. However, the patients in the NA-CHT group of this meta-analysis did not receive Adjuvant hormones, which may have impacted their outcome given the up-regulation of the AR after RT [7]. Moreover, a proportion of patients in the C-AHT did not receive concurrent treatment with RT, as this was a very heterogeneous group including patients with Adjuvant-only ADT.

Despite strong evidence favoring C-AHT, different findings have been reported in large retrospective trials for high-risk prostate cancer. The Cleveland Clinic study by Weller et al. found no significant differences in bRFS, MFS, and OS between patients receiving dose-escalated RT and either NAHT-CHT or AHT at 10 years [49]. Interestingly enough, contrary to Spratt et al.’s meta-analysis [10], no benefit was seen with AHT in this high-risk population. However, similarly to the NRG/RTOG 9413 study, these high-risk patients receiving short-term ADT could be considered undertreated as per the current standard of care.

Conversely, the NCDB study by Lee et al., including high-risk patients treated radically with RT and ADT found a significant OS benefit at 5 years with NAHT vs. AHT (HR 0.86). Although retrospective, lacking data on total ADT duration, and subjected to selection bias, it had the largest sample size (n ≥ 11,000) [50].

Given the potential benefits of the NAHT, the duration of the neoadjuvant period could be a matter of interest. A recent large individual patient data meta-analysis by Kishan et al., mainly including HR and IR PCa, reported no benefit in MFS when prolonging NAHT from 3–4 months to 6–9 months [56]. On the other hand, an NCDB study including 37,606 patients with HR PCa treated with RT plus ADT found improved OS when NAHT was given for 8–11 weeks compared to shorter courses (HR 0.9), but not when extending it beyond 11 weeks [57]. These results suggest that if an underlying benefit to neoadjuvant ADT exists, it could be marred by a delay of over 3 months of the definitive ablative treatment. However, definitive conclusions cannot be made, given that the exact duration of ADT was not collected in these trials.

Conversely, randomized clinical trials on HR PCa have failed to replicate the advantage of short NAHT seen in retrospective studies. RTOG 9413 reported an interaction between the optimal sequencing of ADT and treatment volumes. The limitations of this study were previously discussed and it remains hypothetical that high-risk patients treated with DE-RT, long-course ADT, and pelvic irradiation would see a benefit with Neoadjuvant ADT sequencing. MFS and OS benefits have already been demonstrated when using long-term prolonging ADT (≥18 months) in these patients [20,21,26,27,28,29]. Furthermore, we now have level I evidence supporting the utilization of elective pelvic irradiation [58]. Therefore, the exclusion of patients receiving pelvic RT and long-term ADT in Spratt’s meta-analysis precludes extrapolating their results to HR PCa. This may warrant further study given the intriguing results seen in the NCDB studies using the NAHT approach [50,57]. Moreover, the recently presented SANDSTORM study showed that in patients receiving WPRT, C-AHT was associated with higher rates of distant metastases and the authors support that NA-CHT should be preferred when pelvic RT is indicated [59].

Apart from the clinical outcomes that each sequence has shown, the biological rationales and mechanisms that might support neoadjuvant or concurrent-adjuvant initiation of ADT have been extensively studied in preclinical essays. NAHT has been associated with prostatic cytoreduction and potentially increased radiosensitivity secondary to a decrease in hypoxic tumor zones [3,4,6]. In animal models, neo-adjuvant ADT induces T-cell prostatic infiltration, which can promote apoptosis and radiosensitivity [60]. On the other hand, opposing theories have suggested that NHT may reduce radiosensitivity as it may decrease tumor proliferation when radiotherapy is initiated [61]. Alternatively, following RT there is an overexpression and over-activation of the Androgen Receptor (AR) in prostate cancer cells [7]. This is associated with increased in vitro cell survival and with tumor progression, as the AR promotes the activation of DNA repair machinery genes [7,62]. The benefits from the concurrent/adjuvant ADT sequencing may be explained by an effective suppression of testosterone at a critical period with an up-regulation of the AR, resulting in increased radiosensitivity and greater synergy between RT and ADT. In contrast, an extension of neo-adjuvant ADT without a sufficient length of adjuvant ADT as seen in the RTOG 9413 and the Ottawa 0101 trials would not have had this effect due to the short period of effective testosterone inhibition following RT [7,9,10,51,62]. Conversely, trials with a potentially adequate length of ADT duration after RT, such as the EORTC 22991, may have overcome this issue [38,39].

Beyond the theoretical advantages each ADT sequence offers, important evidence has emerged supporting a reduction in distant metastases with the concurrent-adjuvant approach, predominantly in intermediate-risk prostate cancer patients. MFS is the only intermediate outcome strongly associated with overall survival in localized prostate cancer as reported in a recent meta-analysis of more than 50,000 patients from 75 studies [63]. Concordantly, despite the heterogeneity and limitations seen in Spratt’s meta-analysis, the C-AHT sequencing approach could be considered the preferred option when combining RT and ADT for the curative treatment of localized IR PCa. However, NAHT is also an option for these patients, as recommended in current clinical guidelines [64,65]. This could be especially important when prostate volume reduction is necessary for treatment planning, given the 20–50% prostate size reduction seen after NAHT [66,67,68]. As for the HR PCa, even though these patients were partially included in Spratt’s meta-analysis, the paucity of the data in the literature and the usual long duration of ADT in the adjuvant setting despite the presence of a neoadjuvant component, limits any meaningful interpretation. Therefore, short-NA ADT for 2–3 months should still be the preferred approach, followed by concurrent and long-term adjuvant ADT in HR patients treated with curative RT.

The main limitations of this systematic review include the limited number of randomized trials evaluating Neoadjuvant versus Concurrent ADT sequencing, the lack of randomized trials evaluating ADT sequencing in high-risk prostate cancer patients treated with pelvic RT and long-term ADT, the variable inclusion criteria of trials, at times, not discerning between disease with a more favorable prognosis and the heterogeneity amongst study patient populations, with different proportions of IR and HR PCa. Additionally, the studies identified in this review have major differences in their protocols, especially in treatment volumes, RT dose, length of ADT, and the exact moment of initiation of hormones in relation to radiotherapy, among others. Considering this, it is problematic to make definite recommendations, especially for HR PCa. Nonetheless, this systematic review compiles and weighs the available evidence for optimal sequencing of RT and ADT. It, thus, provides further insight to help to guide clinical practice for patients with IR or HR PCa planning to receive definitive radiation and hormonal therapy.

## 5. Conclusions

The available evidence consistently reports a survival benefit with the combination of RT and ADT in HR patients, favoring courses of 18 to 36 months of ADT, even in the dose-escalated era. In IR PCa, the addition of a short course of ADT to RT improves disease control and may positively impact OS. In the present systematic review, the sequencing of ADT and RT was identified as a clinically relevant point and supports the use of the C-AHT in localized IR PCa. Nonetheless, NAHT starting 8 to 12 weeks before radiation therapy is also an option for this population as stated in current clinical guidelines, especially when prostate volume reduction is clinically relevant for RT treatment planning. As per HR PCa patients, the evidence analyzed in this systematic review does not support concurrent over neoadjuvant hormone therapy. Therefore, these patients should be treated as per standard, with short-NA ADT followed by concurrent and long-term adjuvant ADT.

## Figures and Tables

**Table 1:** Summary of keywords and search results per database.

Database	Search Results	Keywords
PubMed	166	Prostate Cancer, Radiotherapy, Neoadjuvant, concurrent hormone therapy, androgen deprivation therapy, Radiotherapy sequencing, Hormonal Therapy
Embase	372	
Cochrane	1	

**Table 2:** RCTs assessing the combination of RT and HT.

Study	Year	Risk	Treatment	N	Volume/Total Dose	Results
RTOG 85-31 [11,12,13]	1997 2001 2005	Mainly HR N+ 30%	RT alone RT + AHT indefinitely	977	If N+: Pelvis +/− PAo 44–46 Gy Prostate/Prostate Bed 65–70 Gy	Immediate AHT improved LC, MFS, and OS at 10 years
RTOG 86-10 [14,15,16]	1995 2001 2008	Mainly HR N+ 8%	RT alone RT + NA-CHT × 4 months	471	Pelvis 45 Gy Prostate 65–70 Gy	Short course NA-CHT improved BF, DM, and PCSM at 10 years.
EORTC 22863 [17,18,19]	1996 2002 2010	Mainly HR	RT alone RT + C-AHT × 36 months	415	Pelvis 50 Gy Prostate 70 Gy	ADT improved DFS, PCSM, and OS at 10 years. No difference in CV deaths.
RTOG 92-02 [20,21]	2003 2008	Mainly HR	RT + NA-CHT × 4 months RT + NA-C-AHT × 28 months (ADT started 2 months before RT)	1554	Pelvis 44–46 Gy Prostate 65–70 Gy	Long course HT improved MFS at 10 years. OS was also improved in the Gleason 8–10 subgroup.
Crook et al. [22,23]	2004 2009	HR 31% IR 43% LR 26%	RT + NAHT × 3 months RT + NAHT × 8 months	378	If N+ risk > 10–15%: Pelvis 45–46 Gy Prostate: 66–67 Gy	Long course NAHT did not improve DFS at 5 years, except in the HR subgroup.
TROG 96.01 [24,25]	2005 2011	HR 84% IR 16%	RT Alone RT + NA-CHT × 3 months RT + NA-CHT × 6 months	818	Prostate 66 Gy No Pelvis	NA-CHT improved DM, PCSM, and 10-year all-cause mortality when given for 6 months. This was not seen in the 3-months arm.
EORTC 22961 [26]	2009	Mainly HR N+ 8%	RT + C-AHT × 6 months RT + C-AHT × 36 months	970	Pelvis 50 Gy Prostate 70 Gy	Short course ADT is inferior for OS and CSS at 5 years.
PCS IV [27]	2018	HR	RT + NA-C-AHT × 36 months RT + NA-C-AHT × 18 months ADT started 4 months before RT	630	Pelvis 44 Gy Prostate 70 Gy	36-month ADT course is not superior to an 18-month course in OS at 5 years
DART 01/05 GICOR [28,29]	2015 2022	HR 53% IR 47%	DE-RT + NA-CHT × 4 months DE-RT + NA-C-AHT × 28 months	354	Pelvis (optional, treated in 12–16%) Prostate 76–82 Gy	Better OS with a 28-month course at 5 years. At 10 years, clinically relevant OS benefits in HR (NSS) but not in IR.
TROG 03-04 RADAR [30,31,32]	2015 2019 2020	HR 66.3% UIR 31.4% FIR 2.3%	RT + NA-CHT × 6 months +/− Adjuvant Zoledronic Acid × 18 months RT + NA-C-AHT × 18 months +/− Adjuvant Zoledronic Acid × 18 months	1071	Prostate 66 Gy, 70 Gy, 74 Gy or EBRT + HDRB 46 Gy + 6.5 Gy × 3 (EQD2 88 Gy) No pelvis	Better PCSM at 10 years with 18 months of ADT. Zoledronic acid did not impact PCSM.
D’Amico et al. [33,34,35]	2004 2008 2015	Mostly IR HR	RT alone RT + NA-CHT × 6 months	206	Prostate 70.35 Gy No pelvis	RT alone had worse PCSM and OS in men with none/minimal cardiovascular disease at 16.6 years.
RTOG 94-08 [36,37]	2011 2022	HR 11% IR 54% LR 35%	RT Alone RT + NA-CHT × 4 months	1979	Pelvis 46.8 Gy Prostate 66.6 Gy	RT + ADT improved OS in IR but not in LR. The OS curves converge at approximately 15 years.
EORTC 22991 [38,39]	2016 2021	HR 24.8% IR 74.8% LR 0.4%	DE-RT alone DE-RT + C-AHT × 6 months	819	If N+ risk 15% or >: Pelvis 46 Gy Prostate 70, 74, or 78 Gy	RT + ADT improved EFS and DFS, but not OS nor DM at 10 years
PCS III [40]	2020	IR	RT + NA-CHT × 6 months DE-RT + NA-CHT × 6 months DE-RT alone	600	Prostate 70 Gy or 76 Gy No Pelvis	RT + ADT improved BF, PFS, and PCSM compared to RT alone. No difference between 70 and 76 Gy + ADT.
TAP 32 [41,42]	2012 2020	HR	ADT alone × 3 years ADT × 3 years + RT	263	Pelvis 46 Gy +/− 2 Gy Prostate 68–70 Gy +/− 2–4 Gy	ADT + RT improved PCSM at 8 years
RTOG 0815 (Abstract) [43]	2021	IR	DE-RT alone DE-RT + TAS × 6 months	1538	Not reported	RT + TAS did not improve OS but had better MFS, PCSM, and bRFS
PR.3/MRC PR07 [44,45]	2011 2015	HR	Life-long ADT alone Life-long ADT + RT	1205	Pelvis 45 Gy Prostate 65–69 Gy	ADT + RT reduced the risk of death at 10 years.
Ito et al. [46]	2020	HR	RT + NA-C-AHT × 5 years RT + NA-C-AHT × 14 months + iADT thereafter up to year 5	303	Prostate 72 Gy No pelvis	No difference in bRFS, but non-inferiority was not demonstrated for the iADT arm
SPCG7 [47,48]	2009 2016	Mainly HR	TAS × 3 months + Adjuvant Flutamide until death or progression TAS × 3 months + RT + Adjuvant Flutamide until death or progression	875	Prostate 70 Gy No pelvis	RT + ADT improved PCSM and OS at 15 years.

N+ = node-positive, PAo = Para-Aortic, LC = Local Control, MFS = Metastasis Free Survival, HT = Hormone therapy, BF = Biochemical Failure, DM = Distant Metastasis, PCSM = Prostate Cancer-Specific Mortality, DFS = Disease-Free Survival, CV = Cardiovascular, LR = Low Risk, CSS = Cancer-Specific Survival, DE-RT = Dose-Escalated RT, NSS = Non-statistically significant, UIR = Unfavorable Intermediate Risk, EBRT = External Beam Radiotherapy, HDRB = High Dose Rate Brachytherapy, EQD2 = Equivalent Dose in 2-Gy fractions, PFS = Progression-Free Survival, TAS = Total Androgen Suppression, iADT = Intermittent Androgen Deprivation Therapy, bRFS = Biochemical Recurrence Free Survival.

**Table 3:** Summary of the Studies assessing sequencing of ADT.

Trial	Year	Study Type	Risk	Treatment	N	Results	Conclusion
Weller et al. [49]	2015	Retrospective (Single institution)	HR 67.8% IR 32.3%	PORT + NA-CHT × 6 months PORT + AHT × 6 months	515	No difference in bRFS, DM, or OS	Sequencing of ADT does not appear to affect bRFS or DMFS
Lee et al. [50]	2017	Retrospective NCDB	HR	RT + NAHT RT + C-AHT WPRT 63.4% PORT 36.6% ADT Duration was unknown	11,491	NAHT improved median OS by 2.5 months	NAHT sequencing improved OS vs. C-AHT in HR PCa treated with RT + ADT.
NRG/RTOG 9413 [51]	2018	Phase-III RCT	HR (Mainly) IR	WPRT + NA-CHT × 4 months PORT + NA-CHT × 4 months WPRT + AHT × 4 months PORT + AHT × 4 months	1322	PFS and BF were better with NA-CHT + WPRT vs. AHT + WPRT or NA-CHT + PORT.	NA-CHT sequence had better PFS compared to AHT when giving WPRT. In patients treated with PORT, the PFS was worse with NA-CHT than with AHT. There was SS interaction between RT volume and sequence of ADT.
Ottawa 0101 [9]	2020	Phase-III RCT	IR (94–96%) HR (3–5%)	DE-PORT + NA-CHT × 6 months DE-PORT + C-AHT × 6 months	432	bRFS favored the C-AHR arm but not SS.	Possibility of a modest improvement in bRFS or clinical relapse with C-AHT dose-escalated PRT
Spratt et al. [10]	2021	Meta-analysis	RTOG 9413 Ottawa 0101	PORT + NA-CHT × 4–6 months PORT + AHT or C-AHT × 4–6 months	1065	C-AHT/AHT sequencing improved PFS, BF, and DM	Short-term C-AHT/AHT plus PORT improved clinically significant outcomes, including DM.

NCDB = National Cancer Database, WPRT = Whole Pelvic Radiotherapy, PORT = Prostate Only Radiotherapy.

## Data Availability

No new data were created or analyzed in this study. Data sharing is not applicable to this article.
